# Corporate Law Versus Social Autonomy: Law as Social Hazard

**DOI:** 10.1007/s10978-020-09267-7

**Published:** 2020-05-26

**Authors:** Michael Galanis

**Affiliations:** School of Law, University of Manchester, Manchester, M13 9PL UK

**Keywords:** Bureaucracy, Capitalism, Corporate law, Managerialism, Social autonomy

## Abstract

This article argues that corporate law has become the legal platform upon which is erected a social process impeding society’s capacity to lucidly reflect on its primary ends; in this sense, corporate law is in conflict with social autonomy. This process is described here as a social feedback loop, in the structural centre of which lies the corporation which imposes its own purpose as an irrational social end, i.e. irrespective of its potentially catastrophic social consequences. The article argues that resolving the conflict between corporate law and social autonomy is impossible, because it presupposes a change of social paradigm towards one where corporate law as business organisation law has no obvious fit. This questions the social legitimacy of corporate law, signifies its non-permanence and thus opens up the field for seeking radical alternatives in the future.

## Part 1. Introduction

Even the keenest ‘black-letter’ admirer of existing laws would accept that social institutions ought to be open to change, in order to adapt to evolving social goals. However, adaptation is not always possible or at least as straightforward in practice, so that even radical action, such as the recent response to the coronavirus pandemic, may not have a lasting effect or may even exacerbate social hazards. Social institutions and their users can function as forces of inertia, so that society is unable to effectively meet its challenges by redefining its ends. Institutions appear as if they have acquired a dominant agency of their own, even when inertia is socially hazardous.^1^

This article claims that what is currently regarded as standard corporate law has a role in the emergence and persistence of such an inertia problem in our society. Within capitalism, corporate law is a central institutional component of a ‘social feedback loop’—i.e. a self-reinforcing social process—by which the primary corporate purpose, namely wealth accumulation, is amplified and imposed upon society as a supreme and indisputable social end. The mechanics of this loop are fairly simple. Firstly, as an extremely effective device for protecting corporate wealth and for promoting organisational expansion, the basic anatomy of corporate law has found an unprecedented fit with the primary capitalist purpose of infinite wealth accumulation. It has enabled business growth to the extent that economic activity is primarily organised within and between private, manager-controlled bureaucracies.^2^ Thus, corporate law is the legal platform for the bureaucratisation of the economy. At the corporate level, bureaucratic organisation tends to suppress the questioning of objectives. Therefore, once these are internally set by management, they are constantly reproduced by a feedback process within the corporate organisation. Secondly, due to its social dominance, the bureaucratic corporation externalises those privately-set objectives so that they are eventually elevated to social ones and this creates a wider social feedback loop. Corporate law is thus a legal structure for this social process which has become so pervasive that it hinders society’s reflective capacity in relation to its objectives. This capacity is the essential basis of social autonomy, since an autonomous society is founded on the recognition that social goals are endogenously fashioned and therefore subject to social scrutiny without predeterminations.

In other words, by providing the legal foundation for the proliferation of the bureaucratised business organisation, the structural core of the feedback loop, corporate law serves as an institution rendering our society essentially heteronomous, i.e. a society founded on the belief that its primary ends are exogenously and eternally determined. This way, material accumulation acquires eternal validity as a primary social end and its consequences are ignored or marginalised. Corporate law is thus inevitably in conflict with social autonomy and therefore it is a socially hazardous institution.

On this basis and drawing from the theory on bureaucracy and Castoriadis’ social philosophy, this article argues that the social legitimacy of corporate law needs critical re-examination. While engaging in a detailed discussion of alternative organisational forms is beyond this article’s scope, the finding that corporate law is socially hazardous is in itself important: it sets the basic parameters for further research on how business organisation law can be radicalised, in order to dismantle the social feedback loop presented here and sustain social autonomy. The discussion will proceed as follows. The next Part will examine the role and organisational impact of corporate law as a vehicle for business. The third Part will analyse the social significance of these organisational changes to argue corporate law is a socially hazardous institution which reproduces social heteronomy by locking social ends within a capitalist frame. The fourth Part will explore the possibility of instituting social autonomy, as a social ideal, but show that corporate law in its current form can have no obvious place in a society organised on this basis. In the light of these findings, the concluding section summarises the basic parameters of future reform for restoring the social legitimacy of business organisation.

## Part 2. Corporate Law as the Legal Infrastructure of Large-Scale Capitalism

That corporate law is a socially significant institution is revealed by its very long history. The short historical account which follows will show that its role has certainly changed over the centuries to reflect different organisational needs and social conditions, even if its basic anatomy has remained remarkably stable. However, what distinguishes the social function of corporate law within capitalism is that for the first time it is a fully private institution in respect of its purpose, its legitimacy and the interests it protects. This reconception has had a tremendous impact on business organisation and the evolution of capitalism more generally.

### Social Transformation and the Privatisation of Corporate Law

In one of the earliest historical accounts on the corporation, Williston (1888, p. 106) traces its origins initially in primitive associations of individuals who formed entities representing clans, tribes or families.^3^ In classical antiquity incorporated entities were used by groups of artisans or public contractors, and later by church organisations, incorporated medieval guilds, municipalities, charities and hospitals. What Williston emphasises is that in most of their long history the role of corporate entities (and of corporate law) was of a purely public or quasi-public character. They had a wider social role—e.g. monasteries performed to a large extent the role the welfare state has today (Rushton 2001)—or served the purpose of regulating public affairs, whether those were connected to professions, proximity of residence or activity, and so on. Even mercantilist companies conducted trade activities on behalf of the state and this made the distinction between markets and the state quite fuzzy, an important factor for their social legitimacy (Alborn 1998).^4^

Hence, incorporation in the pre-capitalist age was regarded as a scarce privilege and corporate charters were actively regulated by the state which required promoters to show their corporations served the public interest.^5^ With the advent of capitalism all this was to change. As Arnold (1937, p. 186) observes, this era is defined by ‘the prevailing ideal […] of the freedom and dignity of the individual engaged in the accumulation of wealth.’ This is known as the ‘capitalist spirit’, the principal force behind capitalism, which has been taken up by Adam Smith, Karl Marx, Max Weber and many others who have built on their works, in order to trace and explain its true origins. We will return to this later (see fourth Part below), but what matters in this instance is that, in spite of their divergence in other respects, all these strands agree that perpetual wealth accumulation *as an end in itself* is the dominant organisational principle and purpose in capitalist society.

As new social interests emerged carrying and being determined by the capitalist spirit, they also demanded the re-conception of existing institutions and of their relationship with the state. More specifically, business interests required an organisational structure which could serve the attainment of infinite economic accumulation by accommodating increasing scale and complexity. The old legal infrastructure based on trust deeds, contractual partnerships or agency law had served entrepreneurship well as long as business relationships were personal, localised and relatively simple; they were institutions capable of organising small-scale, entrepreneurial capitalism. However, such instruments proved inadequate for large business scale and for attracting investment capital from outside investors (Pearson 2002, p. 862; Taylor 2006, p. 17). So, business organisation law eventually had to turn capitalist by offering a legal structure for large, impersonal and complex business relations.

From an organisational perspective, the corporate structure was an ideal vehicle for large business. While this point will be analytically discussed below, for now it is sufficient to emphasise two things. On the one hand, incorporation facilitated the governance of distant and impersonal relations though the creation of a corporate pool of assets and the delegation of their management to professional directors. Legal recognition of the corporate person as the fictitious entity representing the common business purpose is crucial for this as it provides a fixed owner of business assets and a reference point for corporate governance. On the other hand, it allowed theoretically limitless capital-raising with the issuing of shares as tradable instruments, while also offering investors protection from business risk beyond their control by limiting their liability in respect of corporate debt. As these elements had already been present in pre-capitalist corporations, there was no need to re-invent the wheel; the old corporate structure simply had to be privatised by being assigned to serve the purpose of private wealth accumulation.

Thus, by the mid-1800s, corporate legitimacy solely rooted in public or quasi-public purposes had already come to an end. This demanded the abolition of the old regime of state-controlled corporate charters and the re-conception of the corporation as a privately created and aimed institution. This privatised legitimacy of the corporation inevitably became entrenched in Britain, the heartland of capitalism at the time, with the Joint Stock Companies Act of 1844 and the Limited Liability Act of 1855. This legislation granted general incorporation rights to privately owned businesses and offered shareholders all the protection against business debt previously reserved for quasi-public entities. Both Acts were emblematic legislative attempts engrained in economic liberalism signifying the privatisation of corporate law in exchange for some disclosure requirements towards middle-class investors (Jones and Aiken 1995, esp. p. 69). The latter, thus, became the private enforcers of any remaining (social) scrutiny by being vested with the right to litigate misconduct (Keay 2014, pp. 92–96). In fact, a legal infrastructure of this type for the pursuit of the capitalist spirit was already in place in all major jurisdictions by the mid-1800s (Pistor et al. 2002, pp. 809–810).

In a nutshell, as the private economic sphere expanded, the privatisation of corporate law followed as an inevitable consequence. Contrary to its treatment in other historical accounts as a symbol of (industrial) capital axiomatically (e.g. Ireland 1996, p. 69; Talbott 2016; see also third Part below), the corporation is an ancient organisational form which is, nonetheless, radically adapted within capitalism, in order to serve private wealth accumulation as an overriding social objective whose prevalence is historically distinct. This reconception, however, has eventually led to further social developments, this time promoted, if not triggered, by corporate law.

### Corporate Law (Anatomy) and Planned Capitalism

If the privatisation of corporate law through the liberalisation of the right to incorporate can be regarded as an outcome of social change, *within* capitalism it has led to further social transformation. The offering of the corporation’s organisational features to private business has transformed economic organisation, which is now predominantly ordered by corporate hierarchies and in bureaucratic terms to the extent that capitalism has evolved into something which is not too different from a decentralised and essentially privatised planned system; this is managerial capitalism. This section will focus on how corporate law has supported and promoted this capitalist mutation.

Crucial for this purpose is organisational scale and its association with managerial empowerment. In this respect, Chandler’s (1977, 1990) historical exploration of the interaction between organisational growth and organisational mode has emerged as a standard source.^6^ He demonstrates how advances in transport and communications in the 1800s enlarged markets and created opportunities for business growth in scale and scope. This made mass production and diversification strategies possible for corporations via horizontal and vertical integration. With those strategies emerged a large multidivisional corporate form controlled by a professional managerial bureaucracy with significant immunity from investor scrutiny. Berle and Means (1967) have shown how shareholders as collective owners of control rights were too diffuse and too detached to influence strategic decision-making.

As corporate controllers, managers pursued monopoly or oligopoly rents in saturated markets by restricting competition through collusion, while they simultaneously created new markets by investing in new product development. Marketing and advertising divisions emerged as critical parts in this process by manipulating consumer needs (Marcuse 1964), in order to create and maintain demand. Simultaneously, input costs were controlled via vertical integration and labour costs were contained by the division of tasks which effectively de-skilled and commodified labour wherever necessary (Braverman 1998).

As a result, commodity, capital and labour markets were largely internalised by corporate hierarchies. Simultaneously, through their market share or oligopolistic collusion, managerial corporations were able to influence prices and market conditions. This has been empirically established since the 1930s (Hall and Hitch 1939) and confirms Adam Smith’s (1976, pp. 754–755) aversion towards the corporation as an anticompetitive arrangement; Chandler (1977) has described this as the replacement of the market by the managerial ‘visible hand’. Indeed, competition law has been historically ineffective in containing the concentration of economic power within corporate bureaucracies. For instance, once collusion via cartel agreements became illegal, prohibitions were easily evaded via mergers and acquisitions (Licht 2014, p. 150) so that collusion was also internalised by the merged corporate bureaucracies. The effect has been a turn from the multidivisional incorporated bureaucracy towards the incorporation of divisions as subsidiaries hierarchically controlled by holding corporations (Zey and Camp 1996; Prechel 1997)^7^ and more recently to supply chain networks dominated by a small number of firms in each sector (Humphrey and Schmitz 2001; Witting 2019). Due to this ineffectiveness of competition law in controlling concentration, most markets have become predominantly oligopolistic; a trend that has accelerated since the 1980s (Autor et al. 2017; Griffin 2018; Kamerbeek 2010).^8^

So, with the rise of the managerial oligopolistic corporation, early entrepreneurial capitalism, first in the US and later in other major jurisdictions (Chandler 1990; Hannah 1976),^9^ has mutated into today’s corporate capitalism, a society where markets are internalised and dominated by private (corporate) bureaucracies commanded by virtually unaccountable managers. Corporate controllers’ independence from internal and external pressures is a crucial aspect of this capitalist mutation. It is well recognised in organisation theory that managers’ autonomy unleashes their drive for endless expansion through organic growth and the acquisition of existing or potential competitors (Penrose 1959; Marris, 1998; Odagiri 2008).^10^ This fits perfectly with infinite accumulation as an end in itself. In other words, the managerial corporation is essentially a self-controlled and growth-oriented hierarchy designed to reproduce itself together with its own capitalist ideas.

Managerial autonomy and its effects have not even been contained by the restructuring of financial markets and the economy more generally towards financial capitalism, since the mid-1970s. Financialisation has certainly instigated significant organisational changes in business organisation. On the one hand, it has increased the weight of profit opportunities from asset-price speculation as opposed to production investment (Dore 2008; Epstein 2005; van der Zwan 2014; Tori and Onaran 2018). On the other, it has enhanced the role of investor interests, as the reform of savings systems and their investment in securities markets has concentrated share ownership in the hands of institutional investors and speculators like hedge funds.^11^ Thus, corporate control rights are more tangled with stock market speculation and expectedly managerial corporations are more inclined to emphasise the importance of share valuation in their decision-making. However, this is not a sign of managers’ surrender to external investor pressure.

Instead, corporate bureaucracies have emerged as dominant financial players in sync with other financial speculators. They are actively involved in securities markets by way of mergers and acquisitions involving competitors as well as by profiteering from financial speculation directly.^12^ Managers, especially at the top, rather than being more constrained, have also found a great opportunity in the financialised economic environment, as they can use the stock market to increase their own pay, while they also increase pay-outs to shareholders at the expense of labour interests (Appelbaum et al. 2019; Dünhaupt 2013). They do so by expanding the weight of their share-based remuneration and simultaneously manipulating share prices with extensive share-repurchase programmes (Lazonick 2013; Bergstresser and Philippon 2006), and by brokering deals in the market for corporate control for which they (and shareholders) get heavily rewarded. Thus, if certain observers initially claimed that managerial control would eventually be dismantled and disappear (Jensen 1989), corporate managers have been able to simply align corporate (and their own) goals with the financialised mode of wealth accumulation (Boyer 2005; Froud et al. 2006). What may distinguish early corporate capitalism from its financialised mode is related to the method of economic accumulation—financial speculation has been added as a growing component to wealth accumulation based on mass production—and the distribution of costs (primarily to wage earners) and benefits (asset owners). Both modes, however, remain equally faithful to the leading principle of infinite accumulation as an end in itself (Boltanski and Chiapello 2007; Boltanski and Esquerre 2016) and the managerial corporation retains its position as the primary structure through which this principle materialises.

Thus, modern business practice is typified by the expanding dominance of private incorporated bureaucracies which are largely unconstrained by what is commonly understood as the ‘market mechanism’ either in financial or input and output markets. That being so, some predominantly post-modernist organisation theorists have tried to balance the lack of extra-organisational (i.e. market) pressure on the managerial corporation with what they see as intra-organisational constraints. They emphasise the increasing need for independent ‘knowledge-workers’ as a challenge to manager-controlled hierarchies, which may lead to a post-bureaucratic era defined by organisational methods like project-management and semi-autonomous units (Adler 2001; Fenwick et al. 2019).^13^ However, empirical scrutiny has exposed that such techniques are simply tighter reformulations of hierarchical control relying on the same principles of accountability and surveillance found in traditional hierarchies (Diefenbach and Sillince 2011; Vie 2010; Courpasson and Clegg 2006). Thus, the twenty-first century capitalist organisation remains as bureaucratic as that of the previous century, if not more so. In this sense, corporate capitalism has essentially evolved into an economic system operating according to a decentralised but coordinated plan primarily devised by private bureaucratic organisations run by managers with minimal outside constraints. Hence what is commonly referred to as ‘free-market’ capitalism is a gross misdescription and as real as a unicorn.^14^

The contribution of corporate law to this transformation by which the entrepreneurially controlled firm is replaced by bureaucratised oligopolies has been immense. Without the essential anatomy of standard corporate law all this would have been impossible. More specifically, focusing on the effective implementation of what they call ‘asset partitioning’ Hansmann and Kraakman (2000) have explained the importance of corporate personality and shareholder limited liability as protective devices for the corporate entity’s wealth (‘entity shielding’), on the one hand, and for its investors (‘owner shielding’), on the other. They argue very thoroughly how for large-scale business corporate law, as a form of property law rather than contract law, is necessary for instituting effectively the defensive protection of the corporate entity’s assets from those who control it or from third parties. Without the creation of the fictitious corporate person by operation of the law, asset partitioning would be impossible to enforce against third parties and thus incorporation would be pointless. Ironically, the privatisation of corporate law is now mirrored by the corporate person being offered protection at the highest-level through constitutional law (Grear 2006; Greenwood 2017); this demonstrates how entrenched the corporation has become within capitalism.

The legal ascertainment and fortification of the corporate pool of assets is fundamental for the rise of the bureaucratic corporation, since it offers the proprietary certainty required for enlarging corporate wealth without the risk of misappropriation. Simultaneously, this legal certainty assists in the exploitation of corporate assets for increasing financial liquidity—e.g. via direct borrowing or securitisation—in order to generate and exploit further profit opportunities. It also facilitates the retention and re-investment of profit that has been instrumental in the growth of the managerial corporation. Relatedly, the perpetuity of the autonomous corporate person also matches perfectly the idea of infinite accumulation, because it disentangles the latter from the limitations imposed by the finite human life. Additionally, corporate personality combined with shareholders’ limited liability provide the legal basis for liability management by holding companies through risk externalisation and regulatory arbitrage (Muchlinski 2010; Tweedale and Flynn 2007). This has been instrumental in the expansion of corporate group activity nationally and internationally. Thus, by allowing the transformation of business parts into incorporated divisions (subsidiarisation), corporate law supports endless accumulation not only in a temporal and quantitative, but also in a geographical sense. Simultaneously, limited liability is also facilitative of expansion, organically or via acquisitions, as it allows equity investors to shield themselves from business risk beyond their control—i.e. from the cost of large scale—both directly and via diversification (Blair 2003, p. 439).

More importantly for the current historical moment of capitalism, asset partitioning has also allowed the managerial corporation to adapt to the demands of financialisation. What Ireland (2010) has dubbed as the ‘reification’ of the corporation facilitates the dismantling or combination of business parts as subsidiaries which themselves can be treated as standalone assets. Relatedly, the emergence of the share as an autonomous tradable asset,^15^ a feature concomitant to the recognition of the corporate person, is perfectly aligned with the asset price-based accumulation regime of financialisation. By making possible the tradability of shares (Hansmann et al 2006, p. 1361), corporate personality is a *sine qua non* for the market for corporate control, and an essential element of financialised capitalism.

Additionally, the governance features of the corporation are also finely tuned with and supportive of unbounded wealth accumulation. Blair (2003, pp. 433–434) has highlighted the importance of vesting decision-making with a board of directors as an entity-shielding feature via directors’ accountability towards the corporate person as such.^16^ This point becomes particularly important in large manager-controlled corporations where the board acquires primacy in defining and pursuing the corporate person’s interests condensed in the legal notion of the corporate objective.^17^ This is a theoretically and doctrinally divisive notion in corporate law scholarship, first, due to the question whether the corporate objective ought to include social concerns in some way, and, second, due to its open-ended and malleable legal nature. The latter is no coincidence as the objective’s open-endedness assigns its ultimate formulation to the corporation itself and, more specifically, to whoever controls it. We have seen that before the corporation’s privatisation these issues were more or less settled by the imposition of the public interest by the state as ultimate controller. However, within corporate capitalism the corporate objective is internally determined by managerial hierarchies with very little outside control as shareholders are rather passive monitors (Cheffins 2009). In this context then it should be seen primarily as an entity shielding device against outside interference with managerial autonomy. The latter is further strengthened by the formulation and judicial application of the law governing business judgment, which generally precludes ex-post scrutiny (McMillan 2013; Keay 2006; Deipenbrock 2016). The only differentiation one could make as to how the objective is applied is that, compared to others, in financialised corporations shareholder interests and accumulation through asset-price speculation may carry more weight in decision-making compared to other objectives (Boyer 2000; Mitchell 2001; Jürgens et al. 2002; Stockhammer 2006).

Overall, the privatisation of corporate law has been a precondition for the emergence of bureaucratic capitalism. By protecting corporate wealth and enabling the organisation of large business it serves the purpose of limitless accumulation very successfully; corporate law is the essential legal infrastructure for the bureaucratic corporation. However, the social consequences of corporate law’s privatisation are much deeper and wider. These are explored next.

## Part 3. Structuring the Social Feedback Loop: Corporate Law as Social Hazard

Having demonstrated the contribution of corporate law to the bureaucratisation of capitalism, we will now turn to assessing the broader impact of this transformation. We will show how corporate law has enabled business organisation to reproduce the capitalist spirit to the point where social transformation is now severely hindered by the social structure. To explain this point an appraisal of the essential features of bureaucratic organisation is important.

While bureaucracy is an ancient form of organisation, it is only within capitalism that it became a subject of inquiry with Hegel (1976, paras. 287–297) being the first to discuss its role and character. His focus was on public administration (he wrote prior to the privatisation of corporate law) which in his view has a positive social role as a mediator between the private and public interest. However, he recognised that extensive division of labour carries the risk of bureaucrats losing sight of their social role.^18^ This observation by Hegel had great potential for understanding the extensive social impact of bureaucratisation and was taken up much later by Weber. However, Hegel himself did not expand on it and simply suggested ethical training and social accountability as remedies without being much more specific. As a result, Hegel’s concise analysis has had little influence on modern theory of bureaucracy.

At best, it inspired Marx’s brief critique of Hegel in his early works^19^ where bureaucracy within capitalism is presented as an instrument of domination used by the capitalist class to guarantee its property rights and, thus, exercise its dominance over the working class. In this sense, he treated both state and corporate bureaucracies similarly. However, as Krygier (1985) observes, Marx’s treatment of bureaucratic organisation created insurmountable difficulties for his theoretical adherents who could not explain Soviet bureaucracy’s autonomous power without resorting to paradoxical or contradictory claims; state bureaucrats could not be a social class with independent interests and this contradicted the central Marxist tenet of struggle between capital and labour. In the context of capitalism, this limitation equally applies to corporate bureaucracies, as Marxist theory cannot explain managerial autonomy from capital ownership as managers do not fit neatly into capital or labour. It seems Hegel’s intuition about bureaucrats’ propensity to diverge from their socially designated role was probably correct; it allowed for bureaucracy’s autonomous agency.

Weber was the first to offer a systematic account of bureaucratic organisation and in a way he reconciled the Hegelian concern about bureaucrats’ social conscience with the instrumentality point of Marx. He did this by constructing his ‘ideal type’ of bureaucracy as an analytical tool for organisational practice and by assessing the wider social impact of its widespread use within capitalism. His departure point was that large organisational size is essentially associated with bureaucratisation (Weber 1978, pp. 221 and 224; also Dimock and Hyde 1940, p. 36), which explains how corporate capitalism has emerged as predominantly bureaucratic. As organisational scale increases, personal relationships wane and are replaced as a source and form of ordering by an impersonal hierarchical order. Similar claims have been made by Simon (1976) who finds that large scale and complexity require a hierarchical organisation based on a centralised objective-setting at the top and decentralised operational decision-making through delegation and division of tasks at levels below.

For Weber(ians)—Hegel and Marx would also agree—bureaucratic organisation is grounded on two fundamental and related principles: technique and domination. The former principle refers to managerial expertise in devising ‘rational’ procedural techniques and rules for the pursuit of bureaucratic goals, which are impersonal and independent of other norms that are incompatible with bureaucratic rationality. Managerial tacit knowledge increases efficiency, but at the same time managerial expertise denotes a shift of control towards the highest ranks of managers. In Weber’s own words, ‘[b]ureaucratic administration means fundamentally domination through knowledge’ (1978, p. 225). The second principle, domination, is the disciplinary effect of managerial techniques—primarily through the definition of tasks and objectives as well as through the manipulation of incentives (Edwards 1979, esp. pp. 128–129 and 148–149)—upon those employed in the bureaucracy, who may not share the same goals as those set for their organisation. Thus, bureaucracy is fundamentally an effective incentive alignment instrument for the achievement of hierarchically set goals (Weber 1978, p. 990).

Expectedly, the recognition by Weber of bureaucracy’s potential power over individual social agency has generated an immense literature comparing his efficient ‘ideal type’ with organisational reality. So, some (e.g. Bigley and Roberts 2001; du Gay 2000) have celebrated bureaucracy’s organisational effectiveness, while others (e.g. Dugger 1980) have emphasised its tendency to diverge from the Weberian ideal due to various dysfunctions that allow personality traits (expressed as status seeking, inner circles, personal favours etc.) to affect organisational efficiency. However, even such critics accept the power of bureaucracy over the individual’s mode of rationalising the world which promotes instrumental reasoning over social conscience (Merton 1940).

In fact, this prevalence of instrumental rationality is the very reason for Weber’s own misgivings about capitalist bureaucratisation. He regarded bureaucracy as unable and unsuitable for tackling the political question of social ends (Weber 1978, p. 266 *et seq*.). In this regard, he was very careful in distinguishing *practical* rationality from *substantive* rationality. The former refers to instrumentality, while the latter is concerned with the ‘rationalisation’^20^ of action on the basis of unverifiable value postulates.^21^ As Kalberg (1980, p. 1156) notes, when it comes to systematising action in certain or all life-spheres, such as family, religious, professional life and so on, ‘a radical perspectivism prevails in which the existence of a rationalization process depends on an individual’s implied or stated unconscious or conscious preference for certain ultimate values’. Different individual and social substantive rationalities can easily coexist within the same social–historical moment, even if they have to compete against each other. So what is to be *instrumentally* pursued by the Weberian bureaucracy is to be determined independently and outside of it through a *substantively* rational social function, i.e. by politics. Thus, Weber (1978, p. 222) was careful enough to point out that ideally at the top of the bureaucratic hierarchy ‘there is an element that is at least not purely bureaucratic’. Otherwise, bureaucratic domination over social agency can turn the ‘precision instrument’ into a social hazard.^22^ This is encapsulated in Weber’s renowned ‘iron cage’ metaphor describing the prevalence of instrumental rationality (for the pursuit of economic acquisition) as a result of expanding bureaucratisation in modern society;^23^ Weber had the hindsight of the managerial corporation that Marx or Hegel did not.

Accordingly, Hibou (2015, p. 24 *et seq*.) focuses on the process of abstraction as a necessary function of bureaucratic organisation. She explains how the extensive use of aggregated and quantified information in order to devise standardised rules, practices and targets requires and simultaneously leads to the proliferation of arbitrary abstraction. This (otherwise necessary) form of organisation constructs an artificially static ‘reality’, in order to deal with uncertainty and contingency in the governance process. Governing by aggregation and quantification enables communication between governors and governed through providing a set of stable reference points. But once those are instituted, the illusion of stable certainty also leads the organisation to forget their artificiality and arbitrariness as constructed ‘realities’. The result is a ‘closure of minds’ that can lead to tunnel vision and, as such, stifle reflexive evaluation of established practices, goals or standards. In addition, anything that is not measurable and quantifiable is of lesser or no importance in the governance process, so that substantive rationality is subjected to instrumentality. Similarly, Castoriadis (1997a) critiques bureaucracy on the basis that, instead of merely closing meaning, abstraction ultimately destroys meaning completely.^24^ It does so by reducing qualitative differences and social relations into quantitative ones, so as to eventually produce a measurable value, most preferably in monetary terms, in order to facilitate hierarchical dominance. But this value has no meaning whatsoever beyond a specific irrational belief in its actual validity and usefulness. More explicitly, it has no meaning beyond serving the unverifiable and, thus, irrational goal of economic accumulation.

What is concerning in relation to bureaucratic domination within corporate capitalism is that, as we saw in the second Part above, the managerially-controlled corporate bureaucracy is not simply instrumentally managing, even imperfectly, the implementation of goals set through processes outside of it, as Weber would have liked. On the one hand, the managerial corporation implements the principle of infinite accumulation to perpetuate its own existence in accordance with the capitalist spirit; managerial incentives’ growth orientation has already been mentioned earlier. Simultaneously though, due to its bureaucratic antagonism towards substantively rational goal-setting action, it is unable to reflect upon its own goals and radically alter them if necessary. In this way, it supports a *feedback loop* where economic accumulation requires instrumental reason and instrumental reason perpetuates economic accumulation as a supreme value. Thus, corporate governance issues can only refer to the corporate hierarchy’s instrumental efficiency in achieving this otherwise unverifiable value to the point of irrationality. All this is all too familiar in the context of empirical corporate governance scholarship. It explains, for instance, the non-resolvability of the corporate valuation problem and the impossibility of treating the share price as an accurate reflection of the ‘real’ economic value of production; all these values actually produced are simply arbitrary and fictitious constructions of a particular belief system, and are simply accepted as ‘real’ by investors and managers as a social fact (Haiven 2011). This may fit nicely with the ‘rationalisation’ of the governance process within the framework of economic positivism, but it is absolutely nonsensical beyond it; it is equivalent to organising by (self-)deception (Murphy 2014).

Certainly, this feedback loop of economic instrumentality would be of limited importance, if it was simply contained within the boundaries of business organisation. The ‘iron cage’ of instrumental rationality would not have a generalised impact on society’s substantively rational deliberation and would simply reduce such corporate logic to one of many competing value systems. However, within corporate capitalism we also have the subversion of this social process for two reasons. Firstly and more obviously, the larger the number of people participating in and being governed by corporate bureaucracy, the larger the social influence of the latter’s logic. Indeed, within corporate capitalism labour markets are predominantly organised in bureaucratic terms, which is not surprising given the economic dominance of the managerial corporation (Jacob 2004; also Hamel and Zanini 2016). Secondly, the social power of the latter is also exerted through its extensive channels of influence over policy and culture. The literature demonstrating the excessive levels of large corporations’ socio-political power is immense and need not be reiterated here.^25^ What is noteworthy, though, is that more recently corporate influence has reached extraordinary levels with the use of internet technology for the generation and analysis of ‘big data’ by which even the most private spheres of human agency are placed at the service of corporate profitability. This type of ‘surveillance capitalism’ (Zuboff 2015) is a supercharged form of accumulation which relies on using personal data for predicting and modifying human behaviour in ways that make traditional mass production marketing practices appear as totally innocent and ineffectual. Thus, through these two channels of social dominance operating simultaneously at both micro and the macro level, the managerial corporation imposes its (ir)rationality not only upon itself, but also upon society as a whole. This is of utmost importance from a social goal-setting perspective: the internal feedback loop of corporate bureaucracy, which rationalises corporate action through the dogma of economic accumulation, inevitably emerges as a *social feedback loop*.^26^

This underlies the persistence and intensification of the capitalist spirit over the past two centuries and its further amplification more recently within the financialised version of corporate capitalism. It also explains the conformist character of regulatory reform in response to the global financial crisis a decade ago and, more importantly, to the intensifying ecological crisis.^27^ The bureaucratised private corporation and the financial interests attached to it have become the stratum carrying, instituting and legitimising economic rationality as the ultimate, unquestionable social value system. The same phenomenon is described by Habermas as the ‘colonisation of the world’^28^ by bureaucratic power, effectively denoting the subjugation of Weberian value-based rationality to economic instrumentality in a way that limits critical reflection about values at the individual and social level. In this logic, every aspect of social life can be reduced to quantifiable, monetised values ready to be used for the purposes of economic accumulation. This sustains the spirit of capitalism but also entrenches the corporation’s central role in the feedback loop of economic rationalisation, because it prevents non-conforming and incompatible value postulates from arising even outside the corporate boundaries. Substantive rationalisation is not simply subjugated, but suppressed due to its incompatibility with corporate logic. Thus, social space is created for the capitalist spirit to expand through the generation of a growing number of reductionist postulates like those in empirical corporate governance literature mentioned earlier in this discussion.

As a result, economic accumulation emerges as the ultimate and unchallengeable social value to the extent that society cripples its capacity to reflect upon and re-determine its instituted ends in spite of their irrationality and hazardous consequences.^29^ In other words, the social feedback loop of corporate capitalism is one of social heteronomy where deterministic economic logic appears to be the most prevalent way of rationalising social action. Its structural core, corporate law and the business organisation erected upon it, renders the capitalist spirit a structural concern as opposed to a mere spiritual one. This structural centrality of corporate law also makes its radical reform unlikely without prior social change signified by the prevalence of another spirit. To the extent corporate law impedes such change, it is a socially hazardous institution and, thus, its legitimacy is lost.

## Part 4. The Possibility of Autonomy and the Uncertain Future of Corporate Law

Thus far, we have questioned the legitimacy of corporate law within capitalism on the basis of its instrumental role in the bureaucratisation of business organisation which is now the platform for the social feedback loop imposing economic goals’ primacy as social ends. This ‘iron cage’ effect of corporate law inevitably indicates a very pessimistic prospect about social change. However, the following discussion will show how society cannot be eternally structured as an unbreachable cage of heteronomy by fully suppressing its reflective capacity. However, this also indicates that corporate law’s future as business organisation law is in no way guaranteed.

### The Deeper Roots of Corporate Capitalism as the Path towards Autonomy

To explore the possibility of breaking out of the current heteronomous social setting of corporate capitalism it will be necessary first to discuss in more detail how an autonomous society may emerge. Similarly to the emergence of capitalism in the first place, this would be a process of radical societal change which would necessarily require the uprooting of the capitalist accumulation-orientated paradigm. Thus, the discussion will have to turn back to tracing the latter’s origins. As mentioned earlier, Smith, Marx and Weber provided seminal analyses upon which the three dominant theoretical approaches on the origins of capitalism have been built. Admittedly, the discussion of such important works could only be crude here and does not do justice to their complexity and eminence. It will be shown, however, that none of these threads can adequately explain the rise of capitalism as a radical social paradigm shift and thus fail to offer sufficient guidance on whether and how the heteronomy loop can be broken.

First, the perspective following Smith’s (1976, p. 25) early analysis attributes the drive for capitalist accumulation to human nature and thus considers its pursuit as naturally beneficial. There is a ‘propensity in human nature’, to use Smith’s own words, to engage in self-interested exchanges, in order to promote well-being through wealth accumulation; human nature is thus reduced to the *homo-oeconomicus*. However, this ‘natural’ propensity cannot be easily reconciled with the emergence of economic accumulation as a dominant ideal only relatively recently in human history. Nor does this proposition, so unreservedly adopted in economic theory, offer a convincing basis for its explicit or implicit reference to the natural character of accumulation as a social purpose; it can only be accepted axiomatically and thus has no rational grounding (Hollander 1977). Founded on this axiom, Smith’s perspective cannot offer an explanation for social transformation towards or away from capitalism. At best it resorts to exogenous contingency and this is often the path followed by those adopting capitalism as a natural social ideal.

Thus, North and Thomas (1973) locate the roots of the capitalist spirit in population growth which resulted in market expansion and profit opportunities. However, they do not explain why population growth initiated capitalism in eighteenth-century England, when it could have done so in the Netherlands where a more dramatic population growth was noted two centuries earlier (Wallerstein 1976, p. 276). Similar claims have been based on scientific knowledge expansion in the pre-capitalist era, but again scientific advances have been too linear to suggest a revolution in the strict sense and definitely not unique in history (Kearney 1964). So, even if the problem concerning the axiomatic nature of accumulation could be ignored, the contingency proposition also offers little in relation to explaining social paradigm shifts towards capitalism or any other social system. Additionally, another historical contradiction and irony is that among the initial carriers of the capitalist spirit were the private merchants around Smith’s time who organised their activities as private corporations (McNally 1990, p. 22 *et seq*.). This denies the very essence of free exchange, hence Smith’s hostility towards them and his accusation that they privatised and corrupted mercantilism (Muthu 2008, p. 185). This historical contradiction is of course even more pronounced now that capitalism is predominantly bureaucratic and the corporation is the primary structural pillar of capitalism.

On the other hand, the Marxist strand traces the capitalist spirit to the economic structure of feudalism and reduces it to a reflection of an oppressive mode of production relations (Marx 1909; Brenner 1982). On this basis, there is little fundamentally wrong with accumulation as a (natural) social purpose provided it is socialised;^30^ the standard (but false) prediction of Marx was that this would happen inexorably. So for Marx(ists) the capitalist spirit is mostly an epiphenomenon of structural change from feudalism to capitalism to socialism on a naturally pre-determined path defined by a class struggle between the working and the capitalist class. This struggle is founded upon and ultimately determined by the economic structure of production relations and the commodification of labour (Meiksins Wood 2002, p. 96 *et seq*.). Even if the problem of Marxist historical determinism and its actual refutation by history itself could be ignored, the commodification of labour is not particular to the capitalist era; slavery certainly pre-dates it. Moreover, within corporate capitalism social classes are no longer those observed by Marx and different classes can have similar aspirations and vice versa. This has already been mentioned above in relation to the managerial class; corporate management without ownership rights over capital does not fit the distinction between capitalist and worker (Barbalet 1986; Graeber 2014, pp. 74–75). One could add to this today’s wage-earners who are tomorrow’s pensioners and therefore interested in stock market returns or land and other asset prices. Thus, social change cannot be solely explained by class struggle and one would have to look elsewhere for its roots.

The Weberian strand is more open—though not without its own problems—as to the drivers of social change by referring to various social status groups as carriers of competing ideas (values) some of which may prevail depending on these groups’ relative social power (Wright 2005). It traces the origins of capitalism to the *combination* of technological with cultural factors (e.g. the ‘Protestant ethic’). However, scientific progress is of lesser importance in the emergence of capitalism, because it is cultural factors that determine its uses (Weber 2005, at xxxvii); the emphasis is thus on the world of ideas. For Weber, capitalist accumulation is neither a natural state of affairs (as in Smith) nor a product of economic relations (as in Marx), but the consequence of a particular way of rationalising the world, which prevails over other competing rationalities. In fact, Weber’s major contribution in this respect is not so much his point on the relation between religion and social change, but his emphasis on how religious rationality gradually gave way to instrumental rationality (he called this the ‘disenchantment’ of the world) devoted to the perpetual pursuit of material wealth as the predominant characteristic of capitalism. However, as already mentioned and contrary to Marx’s optimism about social evolution, Weber’s sociological analysis of modern capitalism was quite pessimistic: rationalisation came with the proliferation of the bureaucratic ‘iron cage’ which would become so inexorable that only charismatic leadership outside of it could subject it to a substantively rational process of goal redefinition. History vindicates Weber’s first point, but by relying on charisma as a solution he offers little more than another contingency-based proposition. Charismatic leadership is not guaranteed and, even if it was, it could not ensure a desirable social order; Weber was aware of this problem but could not offer a satisfactory answer (Dow 1978, p. 85).

In this respect Castoriadis’ (1987) theory of radical social paradigm shifts based on the concept of the *social imaginary*, not only responds to this problem in Weberian theory, but also avoids the methodological difficulties and contradictions in the other perspectives described above. His basis is the individual and his emphasis is on the role of imagination, a human capacity that has either been ignored (e.g. in Cartesian rationalism) or rejected as distortive of truth (as in Aristotelian ethics) or subjected to some form of transcendental reason (as in Kant). The prominence of imagination in Castoriadis is more than justified. It is for some time now empirically established by cognitive science that human learning, reasoning and purposive action are entwined with the imagining capacity of the brain (e.g. Hesslow, 2012; Schacter 2012; Gaesser 2012; Stokes 2014; Leahy and Sweller 2008; Dolan 2002). On this evidence, ignoring, rejecting and subjugating imagination as a factor influencing human action in traditional philosophy now seem outdated. Therefore, incorporating it in the study of social phenomena (the law included) has been long overdue and this is exactly what gives currency and force to Castoriadis’ theory on social change and stability. However, as we shall see, this framework offers more than explanations, as it suggests the possibility of instituting social autonomy through a pure conception of democratic polity as a social ideal. Thus, it is not only helpful in explaining (corporate) capitalism’s emergence and appraising the social feedback loop’s ultimate force, but also offers guidance as to how such structures can be dismantled and avoided in the future; this ticks all the boxes for our purposes here.

The starting point for Castoriadis is the subconscious and, in particular, its ability to create imaginary representations *ex nihilo*. This conception of human imagination recognises the psyche’s potential to do more than simply *re-*present external reality that it then simply *re-*produces (Castoriadis 1987, p. 283). Castoriadis calls this psychical capacity the ‘radical imaginary’. This empirically proven cognitive process (Brogaard and Gatzia 2017; Dijksterhuis and Meurs 2006; Dietrich 2004) is the source of radical novelty—creative ‘out of the box’ thinking—rather than the simple re-interpretation or synthesis of already existing and pre-imagined ‘realities’ (Modell 2003).^31^ Interaction with the external world, i.e. perception, relies on imagination which interprets but also gives meaning to this experience. Ultimately the psyche creates its own imagined and therefore subjective representation of the world. It is perhaps accurate to compare this with the subjective understanding of the self and the world described by Weberian substantive rationality; both lead to an open-ended notion of rationality, so as to accommodate different images of the self.

Certainly, Castoriadis (1997b) does not disregard social conditioning and this creative process is ‘not in nihilo or cum nihilo’ (*ibid*., pp. 321–322).^32^ Due to an inherent tendency of the individual as a social being to internalise socially created representations of external reality, the psyche is always socially conditioned. For example, the individual socialised within capitalism will more or less resemble the *homo œconomicus* or some type of personality that fits within the capitalist structure so as to exist within this context. This is indeed the source of the social feedback loop’s power within corporate capitalism. Since, by its nature, corporate bureaucracy is a mechanism for confining meaning within the economic accumulation frame, it effectively restrains the creative capacity of the radical imaginary; its proliferation contradicts individual autonomy. To the extent, however, that this structural power is unable to fully determine the radical dimension of the subconscious, bureaucratic control can never be complete. There will always be a fault-line between the social feedback loop and the individual’s radical imaginary. Thus, imagining a different mode of being, even within capitalism, always remains a possibility which necessarily begins from the individual social agent.

This battleground between socialisation and the radical imaginary is indeed, for Castoriadis, the driver of social change and what therefore created the capitalist spirit in the first place. He links what occurs at the individual level with the emergence of social order by tracing the latter’s roots in the former. It is from the creation of shared images of shared ‘realities’ arising from agents’ co-existence in space and time (the social–historical) that a society is instituted. This shared imagination of society’s individual members as an ‘anonymous collective’ (Castoriadis 1997b, p.322) generates central significations—such as God, the economy, justice or capital—which do not refer to logic or actual reality, but have to refer to other things to signify how society thinks about them. So, within capitalism, capital has to refer to machines, land, work (human resources), shares or, more recently, personal data. Through such second order significations society gives meaning to everything else—e.g. wages, goods, profit, costs and so on—and is held together by referring to them. Thus, a society’s history-specific logic is ultimately determined by its central significations which are unverifiable creations of collective imagination, the ‘radical social imaginary’ (Castoriadis 1987, pp. 358–365). This, as Thompson (1982, p. 664) observes, creates central significations explaining ‘the orientation of social institutions, the constitution of motives and needs, the existence of symbolism, tradition, and myth.’

In this creative process which institutes society, social praxis is of primary importance. Social agents’ spontaneous activity, even if mundane and not necessarily revolutionary, often requires imagining the future and their position in it. From this inevitably originates an infinite creation of new significations (Joas and Meyer 1989) which may not always be compatible with what is already instituted and such contradictions can instigate minor or wider changes. Certainly, social praxis occurs in various domains and among a variety of social groupings where different significatory configurations may emerge. To the extent such groupings see themselves as belonging in the same society they will more or less share that society’s central logic, the dominant imaginary, and remain conformist. Indeed, the transition from entrepreneurial to corporate and financial capitalism can be explained in this way. However, even central significations are not immune from this process of constant flux. Social praxis does not exclude the existence and emergence of other radically different imaginaries carried to various degrees by particular social segments. So there is always a tension within the instituted society, which can eventually bring about radical social change as self-alteration even without clean breaks; Arnason calls this a ‘long revolutionary process’ (Arnason 2001, p. 157; also Ciaramelli 1997). As all societies are subjectively self-instituted, they are also subjected to the forces of change endogenously.

Thus, capitalism can also be explained as the outcome of a particular accumulation of such practice-driven reconfigurations of previous rationalities which gave rise to the capitalist imaginary. The roots of the capitalist spirit can indeed be traced in Weber’s verdict that, in the aftermath of the Enlightenment, the world was ‘disenchanted’ following the retreat—even if not complete—of religious rationalities (Weber 1946, pp. 129–156; also Harrison 2017). This created a vacuum in relation to rationalising life’s finitude, which had to be filled by a phantasy of omnipotence expressed through the domination over nature. In this context, it did not take long until economic rationality became an elite project too, by which social salvation would be attained through the pursuit of material accumulation (Hirschman 1977). Indeed, this is what underlies Adam Smith’s optimism about economic development and is recognised by Mill (1909, pp. 696–699); also Schmidt (2014) or even Marx as the driver of infinite economic accumulation, served by instrumental reason; certainly this is Weber’s verdict too. All this can be attributed to the workings of the radical social imaginary which has instituted what Castoriadis calls ‘rational mastery’ as the central signification guiding capitalist society; he uses the terms ‘pseudo-rational’ and ‘pseudo-mastery’ to denote its imaginary and unverifiable essence. However, this irrationality matters little, since central significations cannot have a rational basis anyway. It suffices that rational mastery has surpassed religion-based salvation so that infinite material accumulation is promoted as a governing social end. As Benjamin pointed out almost a century ago, the emergence of the market or a market ethic as the ‘natural’ arbiter and calculator of social activity is not too different from the metaphysical beliefs which were more prevalent in the pre-capitalist world.^33^ What does matter is that this social imaginary orientates our society, through the process exposed above, by referring and giving meaning in various degrees to all other organisational aspects; an objectively rational basis is completely unnecessary for that to happen.

Indeed, Castoriadis’ major contribution in locating the roots of social framing in the workings of imagination, is that by doing so he has also opened the way towards radical reflection on institutional arrangements and thus towards promoting social change. If transformation towards bureaucratic capitalism can be explained as a product of social imagination, breaking the social feedback loop is also possible without having to resort to the nebulous concept of Weberian charisma. To the extent that the radical (social) imaginary is not constrained, capitalism just like any other previous social order, is also susceptible to incremental change resulting from mismatches between regular praxis and what is instituted. The more such frictions accumulate, the higher the likelihood of generalised questioning of central significations.

Certainty, catalytic events, may even speed up and radicalise social change. For instance, in response to the global coronavirus pandemic, we have witnessed the unprecedented suspension of economic activity imposed by governments, in order to prioritise public health. Simultaneously, the private sector has been mobilised and quasi-nationalised with the substitution of private incomes by state subsidies (Partington 2020). This could signify a moment of radical departure from economic determinism and the supremacy of economic accumulation could be significantly challenged. However, even as such events have been unfolding, the forces of social inertia (and the social feedback loop) have not vanished: the corporate sector—especially big pharmaceutical companies—is still relied upon (and legitimised) as the potential saviour from the pandemic, while even wage subsidies could turn out to be a costly and disguised effort ensuring the survival of established economic rationality. Also, rather striking have been the objections of powerful corporate interests to the prioritisation of public health over the economy, even as coronavirus fatality rates had been accelerating (Reich 2020). Thus, the possibility of a paradigm shift will ultimately depend on whether the epidemic’s social impact prevents a return to the servitude of the capitalist imaginary, once the possibility of sidelining economic accumulation in favour of another social end (e.g. public health) has already been experienced; this had previously been considered ‘irrational’ and impossible. However, the more exceptional a crisis, the more likely the response to it will also be regarded as exceptional. As such, this response may never become systemic, so that social inertia eventually prevails. Still, even if a radical shift away from the capitalist spirit and practice could indeed become a visible possibility after such dramatic events, the next and more important logical question is about the type of any new central signification(s) and its social desirability.

To answer this question, a vital distinction should be made here: while all societies are essentially and always self-instituted, they are not necessarily autonomous, as they tend to conceal their self-instituting capacity. This heteronomy arises when the instituted structure leads to a closure of meaning by suppressing the workings of the radical imaginary, as illustrated in the context of bureaucratic organisations (third Part above). Social heteronomy can be explained on the basis of a central imaginary representation postulating institutions which are based on a rationality presumed objective and extra-social; e.g. God’s will or the market logic as a natural law. This is when the relationship between society and its institutions is ‘turned upside down’, to use Castoriadis’ words, with the former serving the latter (Castoriadis 1987, pp. 91 and 110).

The only remedy and defence against heteronomy is to institute society on the basis of what Castoriadis calls the central signification of autonomy. Effectively, this is a political call for a society that *takes responsibility* for and abides by its own rules, but only for as long as they serve its democratically selected goals by similarly self-reflective and actively responsible citizens in a ‘circle of creation’ (Klooger 2012, pp. 91–92). So, if Weber’s hope for social emancipation rests with charismatic leadership, Castoriadis’ proposition is for a system of institutions promoting lucidly reflective, but necessarily democratic, *practice* as an enduring project of autonomy. Anchoring the radical social imaginary and its institutional manifestation within such a democratic process is the safeguard against the risk of charismatic leaders’ authoritarian subjectivism; not an unknown phenomenon these days.

The infinity of the project of autonomy is indispensable because, contrary to rational democratic deliberation *à la* Habermas (1996, pp. 287–328), for Castoriadis, social outcomes can never be ideal, even if they have emerged through a rational process; procedural rationality principles are also socially determined and value-laden.^34^ All institutions can only be substantively rational and therefore arbitrary to the extent they are founded on unverifiable values which make them always open to radical revision. Social autonomy requires the acceptance of this type of openness and its full enactment includes the revision of social deliberation processes too. Indeed, the social feedback loop problem presented above vindicates Castoriadis’ concern in that current democratic arrangements seem to struggle against corporate power.

The struggle for social autonomy, even in the aftermath of a pandemic or other crisis, is therefore twofold. On the one hand, it is for sustaining radical change against the tide of the social feedback loop of economic determinism for long enough. On the other hand, it is for ensuring that the new structure replacing the old will institute the central signification of autonomy. Unfortunately, the measures adopted in response to the pandemic (general lockdown with the suspension of fundamental freedoms) can hardly be reconciled with autonomy. This risks rendering the social feedback problem analysed in this article even more acute in the future.

### Implications for the Future of Corporate Law

On the basis of the normative position outlined above, the primary social legitimacy criterion for corporate law, as with any other institution, is its compatibility with autonomous reflective action. This open (in terms of corporate legitimacy), but quite prescriptive (in terms of method), criterion necessitates the undoing of the social feedback loop in order to resolve the conflict between corporate law and social autonomy. The question then is if corporate law can indeed accommodate this criterion.

To begin with, that in an autonomous society one would expect to find institutional arrangements promoting democratic deliberation on a substantively rational setting is unquestionable. In such a context, business organisation would be assigned to serve social purposes which are democratically set, without exercising social agency independently of its individual participants. Essentially this presumes that business organisation ought to be non-bureaucratic, otherwise it would undermine individual agents’ reflective capacity and frustrate substantively rational deliberation within its decision-making and governance arrangements more generally.

Nonetheless, the analysis in the third Part above has exposed the impossibility of this within the setting of the managerial corporation and corporate capitalism. We have already established that the social feedback loop and its corporate law foundation is a typical illustration of the inverted social arrangement where society exists to serve its institutions as corporate bureaucracy imposes its goals upon society. The re-imagination of corporate law through its privatisation as business organisation law is the root cause of this problem. Ultimately, through corporate law our society has instituted and reproduces its own heteronomy. Even if not all corporations are managerial and bureaucratic, we have shown how, by its nature as a governance form, corporate law has an in-built propensity towards organisational growth, that is, a bureaucratisation tendency. As such corporate law is a mechanism for confining meaning within the economic accumulation frame and ultimately limits the creative capacity of the radical imaginary; it contradicts autonomy by reproducing the capitalist logic through its re-incarnations as a managerial structure. This is indeed its fatal flaw from the perspective of autonomy and in respect of its compatibility with a society instituted on the basis of that perspective.

Seen in this way, modern corporate law is simply an ideologically charged product of our own society’s self-imposed imaginary. In other words, corporate law, the legal foundations of the social feedback loop, can only be explained and justified by adhering to the heteronomous capitalist logic and the ideology that follows and solidifies it. As observed by Lefort (1978, p. 296), ideology conceals the imaginary nature of central significations through ‘the linking together of representations which have the function of re-establishing the dimension of society ‘without history’ at the very heart of historical society’. The significance of this is that it crystallises meaning by making issues which are essentially social, political and therefore temporary look as if they are simply of a technical nature with little normative background (Thompson 1982, p. 672) and of eternal legitimacy. The proliferation of pseudo-scientific mathematical modelling in the justificatory framework for current corporate law doctrine is simply another sophisticated tool concealing the ideological roots of corporate law with a pseudo-scientific gloss (Chen and Hanson 2004).

Lifting this technocratic facade would simply reveal that corporate law is a deeply political instrument and would open the door towards re-subjecting it to political (i.e. democratic) deliberation about its use. Indeed, this way of treating corporate law could open the door to democratic reflection which could resolve its conflict with society. However, this re-politicisation would contradict its very nature as an institution serving private business interests, i.e. infinite private accumulation as a social end. After all, it was privatised in order to facilitate the supremacy of private economic interests over the public interest through reducing the latter to the former. If this subjugation has not been problematic within capitalism, its questioning is equivalent to doubting the very foundations of our society so long as it is guided by the capitalist imaginary. The more business activity diverges from what is regarded as the public interest (as it increasingly seems to be the case) the trade-off between business organisation and social objectives becomes more pronounced and this delegitimises the former. As mentioned above, the temporary suspension of capitalist accumulation could indeed instigate radical social reflection on the social role of corporate law, but inertia could yet again prevail after the pandemic’s containment; e.g. the focus could be targeted towards economic ‘rebuilding’ along conventional lines but with higher intensity. Indeed, the first signs of the feedback loop’s further entrenchment are already visible, as among the post-coronavirus reforms considered by the British government is the introduction of manager-friendly corporate insolvency rules (Partington and Stewart 2020).

If the legitimacy problem of business organisation is to be resolved, its governing law ought to take a radically different turn by imposing limits on the concentration of social power by private organisations outside and above the reach of democratic scrutiny.^35^ Otherwise, even if the social feedback loop’s force could be temporarily suspended by social crisis to allow for the emergence of a new social logic, corporate bureaucracy would at best mould it to fit its own interests (even if radically new) as another type of ‘iron cage’ or it could revert and re-impose the default business purpose of endless accumulation yet again. As explained earlier, the root of corporate law’s socially hazardous nature is its orientation towards accumulation which leads to increasing scale, bureaucratisation and the freezing of meaning in economic terms. Thus, one of the preconditions for engraving autonomy in the social structure is to socialise business organisation through the imposition of an organisational limit to its scale. This would dismantle the heteronomy loop by removing the dominant agency that business organisation currently has acquired, which subjugates social scrutiny. It would also prevent the colonising effect of business practice and ideology outside of its strict limits in other sectors of public and private life. Business organisation would thus become more of a receiver of social significations and more adaptive to social purposes. What is clear from the analysis so far though is that corporate law as a structure for business organisation has no evident fit in a social context of this kind—its ‘legitimacy’ exists only in a heteronomous social setting. Therefore, the use of corporate law as business organisation law ought to be marginalised and replaced by structures respectful of autonomy. In fact, if a prediction can be made, this could be inevitable to the extent that capitalist accumulation has reached its natural limits and inexorably leads to societal adaptation towards a post-growth era.^36^ This is indeed an insurmountable challenge to capitalist society’s central signification, the phantasy of ‘mastery over nature’, whereas the coronavirus pandemic’s impact could also fertilise the ideological debate. However, more effort along the lines suggested in this Part will be necessary if social autonomy is to be instituted, so corporate lawyers’ social role is specific and significant.

## Part 5. Conclusion

In spite of corporate law’s social impact discussed here, we have established that social paradigm change is after all inevitable, since our social order is subjected to the radical imaginary’s creative forces and therefore can never be eternally set. However, it matters little whether the heteronomy loop of corporate capitalism can be temporarily broken, if this breaking does not lead to an autonomous society and is replaced instead by some other heteronomous social arrangement. Thus, what matters most is that social focus and therefore academic effort should be on replacing the current relation between society and its structure with a different one instituting the principle of autonomy. Inevitably, within this frame our business organisation law ought to look very different to how it currently does and its legitimacy must be grounded on its capacity to accommodate self-reflection, rather than being based on some other ‘objective’ (ir)rationality.

As a first start in this direction, even if this is a painful process for corporate lawyers, we should acknowledge that corporate law’s social legitimacy has already been lost. From this acknowledgment begins the responsibility to re-envisage business organisation law in radically different terms, if it is to cease being an institution of hazardous social inertia. The paradigm of social autonomy constitutes a good general guide in this process which undoubtedly will have to be dynamic and perpetual. Even if working out the detail of what business organisation law ought to be is beyond the endeavour of this article, the discussion above has revealed one major aspect of corporate law causing its legitimacy problem as a hazardous institution of heteronomy, namely its inherent orientation towards endless accumulation. Law reform in this area could then start by actively promoting the marginalisation of corporate law in the structuration of business organisation. Positive action along these lines could involve the promotion of non-corporate legal structures for non-hierarchical organisation which can accommodate autonomous agency better. To the extent radical social change is at least imaginable (if not inevitable), the ‘black-letter’ corporate lawyer (an increasingly imaginary figure) would then need to be prepared for the marginalisation of the private business corporation. Delaying this could eventually backfire as the increasing divergence between corporate and social interests may eventually take a more authoritarian or violent turn, and the coercive reaction to the coronavirus is not too promising.

## Footnotes

1 The persistent denial of the trade-off between economic objectives and social sustainability or the inadequacy of respective action illustrate this problem (Hoffman and Ventresca 1999; af Rosenschöld et al. 2014). Even a dramatic global pandemic may not be enough for sustained systemic change (see fourth Part below).

2 ‘Bureaucracy’ refers to the organisation designed as a top-down hierarchical decision-making and accountability structure.

3 See also Hansmann et al. (2006, pp. 1360–1361) discussing the Roman *societates publicanorum*.

4 Indicatively, the East India Company’s original charter granted by Elizabeth I was conditional on the venture being regarded as profitable for the Crown.

5 Monastic entities were somewhat exceptional in this respect as they were the ones that pioneered incorporation in Britain before the state began to exercise its control over it (Seymour 1903).

6 Even his critics focus on his interpretation of historical facts as an efficient transformation rather than on his descriptive accuracy (e.g. see Roy 1997).

7 The validation of single-shareholder companies in English law since *Salomon v Salomon & Co Ltd* [1897] AC 22, allowed the creation of subsidiaries relatively early (Chapman 1985). In the same fashion, initial prohibitions of holding companies in the US were gradually lifted in the 1880s (Butler 1985, pp. 161–163). Regarding German corporate groups, Fohlin (2005, p. 228) reports that ‘in 1904, less than 1 percent of [corporations] held nearly a quarter of the corporate capital stock’.

8 On the dilemmas faced by regulatory authorities related to cartel versus merger control see LeClair (2011, esp. pp. 151–154).

9 For a more recent claim that the managerial corporation was already dominant in the UK by the beginning of the twentieth century see Foreman-Peck and Hannah (2012).

10 See also Guglera et al. (2012) linking merger waves to managerial incentives.

11 This shift is more noticeable in the US and the UK, but similar trends are also noticed elsewhere including social-market economies as that in Sweden (OECD 2018).

12 Indicatively, Apple Inc. owns what is effectively the world’s largest investment fund, Braeburn Capital, managing financial assets worth over 250 billion US dollars.

13 This misconception by organisational theorists also (mis)led prominent legal theorists to speculate that new business necessities could transform capitalism into a de-bureaucratised, network-based and inclusive system reflecting the so-called ‘knowledge economy’ (e.g. Unger 2019).

14 On this point see also Crouch (2011) and Hamel and Zanini (2016). In fact, managerial employment never stopped increasing, even since the 1980s, the era when downsizing and de-bureaucratisation strategies were supposed to dominate (Gordon 1996; Goldstein 2012).

15 For a detailed historical account see Ireland (1996).

16 Indicative of corporate law’s approach towards board autonomy is that directors’ duties enforcement is essentially hindered both procedurally and practically (e.g. Reisberg 2007).

17 The objective is essentially tied to the promotion of the company’s success as a going concern (e.g. Keay 2011, p. 10; Dignam and Galanis 2009, pp. 57–60); see also Attenborough (2009) on an alleged doctrinal closure since the introduction of s.172 of the Companies Act 2006.

18 A similar claim was made by Mill (2011) who recognised the bureaucratic tendency towards becoming a ‘pedantocracy’.

19 See Marx (1970) and Marx and Engels (1974).

20 This term is in inverted commas here is because, for Weber, substantive rationalisation is never based on transcendental, universal or absolute values; values are not scientifically demonstrable, but grounded on plain individual and thus social belief in them.

21 Kalberg (1980) offers a thorough review of the use of the concept of rationality in Weber’s writings.

22 In a more recent critique, Graeber (2015) discusses bureaucracy’s irrationality as an organisational mode fostering ‘stupidity’.

23 Weber (2005, p. 123) used the term to describe the capitalist order which ‘is now bound to the technical and economic conditions of machine production which today determine the lives of all the individuals who are born into this mechanism, not only those directly concerned with economic acquisition, with irresistible force’. For a recent analysis on the meaning of the Weberian ‘iron cage’ see Douglass (2016).

24 On this point see also Murphy (2014) and Graeber (2018). Bureaucratic meaninglessness is now widely perceived in the workplace. Indicatively, a recent public survey by YouGov (2015) has revealed that 37% of working adults in the UK thought their job made no meaningful contribution to society, with another 13% not knowing whether their job did or did not.

25 For a review see Hillman, et al. (2004). On the primary channels of corporate political influence see Barley (2007) and Sitkoff (2002). For a claim that corporations have now assumed public functions so as to become political agents see Crouch (2011) and Scherer et al. (2014); see also Maclean et al. (2010) for an empirical study confirming the social power of small corporate elites in the form of material and symbolic domination.

26 This conclusion is at odds with Crouch’s (2011) conclusions contemplating that social critique of corporate activity can have a real impact. The argument here is that the impact of social critique on corporate reform is significantly stifled by the social feedback loop of bureaucratic capitalism.

27 The thorough review of corporate law evolution by Johnston (2017) clearly demonstrates the conformist nature of legal reform, which increasingly excludes societal concerns by focusing managerial accountability on the prioritisation of financial (shareholder) interests. Similarly, the soft approach towards engagement expectations from institutional shareholders with otherwise pressing environmental concerns in the UK Stewardship Code 2020 can also be explained by prevailing economic rationality (Katelouzou 2019). In fact, the judiciary too seems to have internalised the capitalist spirit as illustrated by the High Court ruling in *R (on the application of People & Planet) v HM Treasury* [2009] EWHC 3020 Admin. I am indebted to one of the journal referees for this point.

28 Habermas (1984, pp. 154 and 196) describes the process as one where ‘economic and bureaucratic spheres emerge in which social relations are regulated only via money and power’. Similarly, in his influential critique Marcuse (1964) focused on the rise of the consumerist culture driven by the preference manipulation strategies of the oligopolistic corporation as a social control mechanism imposing the capitalist spirit of irrational accumulation.

29 Discussing the hazards of capitalist accumulation is beyond this article’s scope. However, its irrationality is highlighted by Piketty’s (2014) discovery that even the narrow purpose of accumulation, namely social economic prosperity, is not achieved due to rising income inequality; the so-called ‘trickle-down’ theory has no solid empirical grounding (Akinci 2018). I am grateful to one of the referees for this argument.

30 Even Arrighi’s (1994, 2007) otherwise elegant analysis of capitalism suffers from this, as it ultimately proclaims state-controlled accumulation (Chinese-style) as a desirable social model.

31 See also Adams (2005, p. 35) referring to the transcript of a discussion on this point between Castoriadis and Ricoeur.

32 As Castoriadis (1997b, pp. 321–322) explains, this psychical capacity is ‘an a-causal vis formandi. A-causal does not mean “unconditioned” or absolute, ab-solutus, separated, detached, without relations’. For more nuanced interpretations of the radical imaginary see also Adams (2005).

33 Benjamin (1996, p. 288) famously described the structure of capitalism ‘not merely, as Weber believes, as a formation conditioned by religion, but as an essentially religious phenomenon’.

34 On Castoriadis’ critique of Habermas’ rationality claim see Kalyvas (2001).

35 This would also alleviate the problems faced by competition law, since a scale-constrained business organisation would be unable to attain a dominant position (see above notes 7–8 and text).

36 While influential studies like Gordon (2016) show GDP growth is anything but guaranteed, there is no doubt that environmental degradation resulting from economic activity has for some time now been generating increasing social movement pressures against the status quo (Rowell 1996).
